# Removal of Heterologous Sequences from *Plasmodium falciparum* Mutants Using FLPe-Recombinase

**DOI:** 10.1371/journal.pone.0015121

**Published:** 2010-11-30

**Authors:** Ben C. L. van Schaijk, Martijn W. Vos, Chris J. Janse, Robert W. Sauerwein, Shahid M. Khan

**Affiliations:** 1 Department of Medical Microbiology, Radboud University Nijmegen Medical Center, Nijmegen, The Netherlands; 2 Leiden Malaria Research Group, Department of Parasitology, Center of Infectious Diseases, Leiden University Medical Centre, Leiden, The Netherlands; INSERM U1016, Institut Cochin, France

## Abstract

Genetically-modified mutants are now indispensable *Plasmodium* gene-function reagents, which are also being pursued as genetically attenuated parasite vaccines. Currently, the generation of transgenic malaria-parasites requires the use of drug-resistance markers. Here we present the development of an FRT/FLP-recombinase system that enables the generation of transgenic parasites free of resistance genes. We demonstrate in the human malaria parasite, *P. falciparum*, the complete and efficient removal of the introduced resistance gene. We targeted two neighbouring genes, *p52* and *p36*, using a construct that has a selectable marker cassette flanked by FRT-sequences. This permitted the subsequent removal of the selectable marker cassette by transient transfection of a plasmid that expressed a 37°C thermostable and enhanced FLP-recombinase. This method of removing heterologous DNA sequences from the genome opens up new possibilities in *Plasmodium* research to sequentially target multiple genes and for using genetically-modified parasites as live, attenuated malaria vaccines.

## Introduction

The genomes of several different *Plasmodium* species are either completely sequenced, or near to completion. This includes those of the most important human malaria parasites, *P. falciparum* and *P. vivax*, as well as three closely related rodent species; *P. chabaudi*, *P. yoelii* and *P. berghei* [1], [2], [3], [4]. Comparative analyses of *Plasmodium* genomes and genomes of other organisms have greatly improved the identification and assignation of putative functions to *Plasmodium* genes, and these analyses have revealed that about 50% of malaria parasites genes cannot be assigned a function by homology and it is therefore likely that many of these genes perform functions that are unique to *Plasmodium.* In the absence of efficient forward genetic screens, reverse genetics, specifically targeted gene deletion and phenotype analysis, is currently the front line methodology to study *Plasmodium* specific gene function [5]. Currently, the permanent removal of genes from the human parasite *Plasmodium falciparum* requires the targeted integration of plasmids into the genome by double cross-over homologous recombination. This approach uses a ‘positive-negative’ selection strategy and results in the introduction of drug resistance-markers into the genome[6]. Specifically, transgenic parasites that have the targeting construct integrated by single-cross over recombination are first selected using one of a limited set of resistance markers and drug combinations[5], [6], [7], [8]. Subsequently, these parasites are subjected to ‘negative’ drug selection to select for mutants that have permanently removed the gene of interest by an internal double cross-over recombination event[6], [9]. The limited number of resistance markers in *P. falciparum* severely compromises the possibilities for sequential genetic modifications. As a result no *P. falciparum* mutants have currently been reported where two or more genes have been targeted by sequential transfection procedureshttp:///. Therefore, there is a need for methodologies that allow for the generation of mutants which lack resistance markers and permit successive gene deletions within the same genome.

A recent development using reverse genetics in rodent parasites has been the generation and analysis of ‘attenuated’ parasites engineered through gene-deletion. These genetically attenuated parasites (GAP) can either become developmentally arrested subsequent to invasion of liver cells [10] or infections with those GAPs that are associated with a marked decrease in the virulence in the host[11], [12], [13]. A number of these lines are now being tested and used in research aimed at developing malaria vaccines that consist of attenuated parasites. The translation of such genetically modified parasites into human vaccines may require the removal of resistance markers from the parasites genome. Specifically, multiple gene deletions may be necessary to reach complete attenuation and removal of resistance markers is essential in light of regulations governing the use of genetically attenuated organisms in vaccines[14], [15]. Here we report on the development of an efficient FLP recombinase system that in combination with the positive-negative drug selection strategy permits the generation of *P. falciparum* gene deletion mutants lacking resistance markers. The yeast FLP recombinase recognizes a 34 nucleotide FLP recognition target (FRT) site and excises any intermediate DNA sequences located between two identically oriented FRT sites (referred to FRTed sequence)[16]. The FLP/FRT system has been previously applied in *Plasmodium* for generation of a ‘conditional knock-out’ system for deleting genes from the rodent parasite *P. berghei* in mosquito stages[17], [18], [19]. However, using the FLP/FRT system to efficiently delete genes in blood stage parasites has not been reported. In this paper we now describe the removal of the resistance marker using a 37°C thermostable enhanced FLP recombinase from a parasite in which the neighbouring genes *p52* and *p36* were deleted. This mutant is actively being pursued as a potential GAP for use in humans, using the standard approach of generation gene deletion mutants[20], [21]. The ability to remove resistance markers from the *P. falciparum* mutant genome will be important not only for research into parasite gene function but also for generating genetically-modified parasites that may serve as live, attenuated malaria vaccines.

## Results

### Generation of a gene deletion ‘FRT’ targeting construct for *P. falciparum*

**Figure 1:**
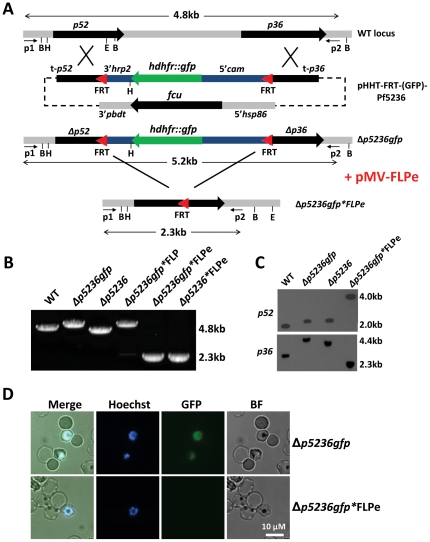
FLPe mediated excision of resistance markers from *P.falciparum* gene deletion mutants. (**A**) Schematic representation of the genomic locus of wild-type (WT), gene deletion mutant Δ*p5236gfp* before and after removal of the *hdhfr::gfp* resistance marker. The construct (pHHT-FRT-(GFP)-Pf5236) for targeting deletion of the *p52* and *p36* genes contains the two FRT sequences (red triangles) that are recognized by FLP. P1, P2: primer pairs for LR-PCR analysis; B (*Bcl*I),H (*Hin*dIII), E(*Eco*RI): restriction sites used for Southern analysis; *cam:* calmodulin*; hrp:* histidine rich protein*; hsp:* heatshock protein*; fcu:* cytosine deaminase/uracil phosphoribosyl-transferase; *pbdt: P.berghei dhfr* terminator. (**B**) Long range PCR analysis of genomic DNA from WT and mutants Δ*p5236* and Δ*p5236gfp* before and after transfection with constructs containing FLP or FLPe, confirming removal of the *hdhfr::gfp* resistance marker in FLPe-transfected parasites. See A for location of the primers p1 and p2 and the expected product sizes (i.e. WT, 4.8 kb; Δ*p5236*, 4.6 kb; Δ*p5236gfp*, 5.2 kb; Δp5236gfp*FLPe and Δp5236*FLPe, 2.3 kb). (**C**) Southern analysis of restricted genomic DNA from WT and mutants before and after transfection with constructs containing FLPe, confirming removal of the *hdhfr::gfp* resistance marker in the FLPe-transfected Δ*p5236gfp* mutant. Upper panel: DNA was digested with *Hin*dIII/*Eco*RI (probed with *p52* targeting sequence); Lower panel DNA digested with *Bcl*I (probed with *p36* targeting sequence). (**D**) Analysis of GFP expression in mutant Δ*p5236gfp* before and after transfection with constructs containing FLPe, confirming removal of the *hdhfr::gfp* resistance marker in the FLPe-transfected parasites.

In order to permit removal of resistance markers from the genome of *P. falciparum* during blood-stage culture, we introduced 2 FRT sites into the standard positive-negative transfection construct (pHHT-FCU)[9] along with gene integration sequences designed to simultaneously target the *P. falciparum* paralogous genes, *p52* and *p36*, resulting in the construct, pHHT-FRT-Pf5236 (Figure 1A). The genes *p52* and *p36* are a closely related and paralogous pair of genes which are located in tandem on chromosome 4 in the *P. falciparum* genome, separated by only 1.4 kb[20], [21], [22], [23], [24]. In the pHHT-FRT-Pf5236 construct the FRT sites have been positioned to flank the two *p52/p36* gene-targeting regions in an identical orientation (Figure 1A). This orientation should enable FLP-mediated excision of the *hdhfr*-resistance cassette located between the FRT sites. We further modified this vector by replacing the *hdhfr* resistance marker for a *hdhfr::gfp* fusion gene, thereby producing the construct pHHT-FRT-(GFP)-Pf5236 (Figure 1A). The *hdhfr::gfp* fusion gene permits both the selection of transformed parasites by WR99210 treatment and the visualization of transformed parasites by fluorescent microscopy.

### Generation and characterization of FRT containing Δ*p5236* and Δ*p5236gfp* parasites

The constructs pHHT-FRT-Pf5236 and pHHT-FRT-(GFP)-Pf5236 were independently transfected into *P. falciparum* blood stages using electroporation [25]. In these experiments double-cross over gene deletion mutants were selected (referred to as Δ*p5236* and Δ*p5236gfp*) by standard positive -negative selection using the drugs WR99210 and 5-FC respectively [9]. The correct integration of the two constructs into the genome of parasites that had undergone positive and negative drug selection was analysed using an adapted long-range PCR (LR-PCR) method and Southern Analysis. The ability to amplify >5 kb DNA sequences by LR-PCR permits us to now rapidly screen the genotypes in parental populations of transfected *P. falciparum* parasites (see Material and Methods for details of the optimized LR-PCR method). Using both LR-PCR and Southern analysis we confirmed that deletion of *p52/p36* by double cross-over integration of the targeting constructs had occurred (Figure 1B, C). Next, Δ*p5236gfp* parasites were analyzed by fluorescence microscopy and all parasites displayed GFP-expression (Figure 1D, top panels).

Unlike in conventional *P. falciparum* gene deletion transfection experiments parasite cloning was not performed at this stage and we proceeded directly with the next step, specifically the removal of the resistance marker between the 2 FRT sites from the genome of Δ*p5236* and Δ*p5236gfp* parasites. For excision of the FRTed sequence, we generated two additional plasmids for transient expression of FLP after episomal transfection into the FRT-containing parasites.

### Generation of FLP recombinase containing plasmids and removal of resistance genes from Δ*p5236* and Δ*p5236gfp* parasites

Two plasmids were generated that contain an FLP recombinase under the control of the *hsp86* promoter and the *blasticidin-S-deaminase* (*bsd*) resistance marker (see Material and Methods and Figure S1A). The first construct, we term pMV-FLP, was constructed by inserting the standard FLP encoding gene under control of the constitutive *P. falciparum hsp86* promoter (HSP86-FLP-PBDT) into the pBSII-KS+ plasmid along with the positive selection marker *bsd*-cassette under control of the *P. falciparum hrp3* promoter (5′HRPIII-BSD-3′HRPII) derived from pCMB-BSD[7]. The second construct, pMV-FLPe, was essentially identical to pMV-FLP except that the standard FLP encoding gene was replaced by a gene encoding FLPe. This plasmid was termed pMV-FLPe. Whereas FLP, being derived from yeast, has an enzymatic optimal temperature around 30°C[26], FLPe is a 37°C thermostable enhanced allozyme of FLP recombinase[27].

The Δ*p5236* and Δ*p5236gfp* mutant parasites were transfected with either the FLP or the FLPe containing plasmid and transformed parasites were selected by blasticidin treatment[7]. The transformed parasites became apparent in the cultures between day 6–13 after transfection. Interestingly, after transfection with these constructs we observed that both these enzymes, FLP and FLPe, had an effect on growth of asexual blood stage parasite (for more details please see Figure S1B and legends).

When we analysed the genotype of FLP-transfected parasites after blasticidin selection we found evidence that in a small percentage of parasites DNA sequences, including the resistance genes, between the FRT sites (i.e. ‘FRTed’ sequence) had been removed. LR-PCR of Δ*p5236gfp* parasites, after FLP plasmid transfection, amplified two fragments of 5.2 kb and 2.3 kb, consistent with the retention of the selection marker and FRT-mediated recombination, respectively (Figure 1B). The 2.3 kb fragment was cloned and sequenced which confirmed the correct excision of the FRTed sequence (data not shown). However, the 2.3 kb PCR fragment was very faint whereas the 5.2 kb fragment of the region containing the FRTed sequence was rapidly amplified (Figure 1C), indicating that most parasites still contained the FRTed sequence. This was confirmed by fluorescence microscopy as more than 99% of the FLP-transfected parasites were GFP-positive. As the optimal temperature for FLP is 30°C[28], we also cultured the FLP-transfected parasites at 30°C for intermittent periods (4–48 hours). However, at 30°C no increase in removal of the FRTed sequences was detected as demonstrated by a similar high proportion (>99%) of GFP-expressing parasites (data not shown).

In contrast to the FLP-transfected parasites, no GFP-positive parasites were visible in the parasite populations after transfection with the FLPe-containing plasmids (Figure 1D), indicating the efficient removal of the FRTed resistance marker cassette. Further, LR-PCR revealed only the 2.3 kb band, consistent with full removal of the FRTed sequence and we were unable to detect the 5.2 kb fragment of parasites that retained the FRTed sequence. These results indicate that FLPe mediated recombination between the FRT sites is highly efficient resulting in removal of the FRTed sequences in nearly 100% of the parasites. FLPe-transfected parasites were cloned by the method of limiting dilution and Southern analysis of cloned parasites confirmed correct excision between the FRT sequences, resulting in excision of drug selectable marker and *gfp* fusion cassette (Figure 1C). These results demonstrate that the FLPe-recombinase system permits the efficient generation of gene deletion mutants lacking resistance markers.

### Analysis of drug sensitivity and gametocyte production in mutants after FLP-mediated removal of resistance genes

We next analyzed if the transfected parasites had retained their capacity to produce gametocytes. The loss of gametocyte production has been reported to frequently occur during prolonged periods of in vitro cultivation and manipulation of *P. falciparum* asexual blood stages[29]. A stable gametocyte production is of particular importance for Δ*p5236* parasites, which are being developed as potential attenuated sporozoite vaccines. For both Δp5236*FLPe and Δp5236gfp*FLPe, gametocyte production, as determined by counting stage II and stage IV-V gametocytes, as well as male gamete formation as determined by counting exflagellation centres was comparable to wild-type (NF54) parasites (Table 1).

**Table 1:** Gametocyte production and male gamete formation (exflagellation) of wild type (WT) and mutants, Δ*p5236* and Δ*p5236gfp*, before and after FLPe action.

Parasite line	No of gametocytes stage II (range)^1^	No of gametocytes stage IV-V (range) 1	Exflagellation^2^
**WT**	10 (2–24)	50 (39–58)	++
**Δ** ***P5236***	11(4–17)	52(44–59)	++
**Δ** ***P5236GFP***	11(8–15)	49(8–65)	++
**Δ** P5236*FLPe	11(6–15)	67(54–79)	++
**Δ** P5236GFP*FLPe	9(2–23)	62 (50–72)	++

1 Number of gametocytes per 1000 erythrocytes counted in Giemsa stained thin blood smears.

2 Exflagellation centers counted in wet mounted preparations of stimulated gametocyte cultures at 400x magnification using a light microscope; ++ score  = >10 exflagellation centers per microscope field.

**Figure 2:**
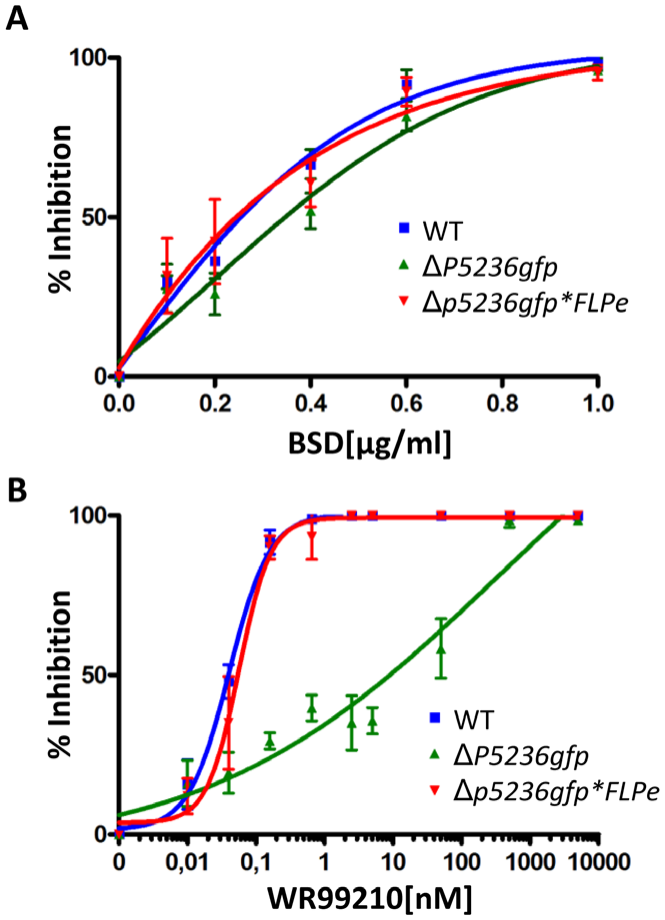
Drug sensitivity of wild type (WT) and Δ*p5236gfp* parasites. Drug sensitivity to (**A**) blasticidin and (**B**) WR99210 of blood stages of WT and mutant Δ*p5236gfp* parasites before and after transfection with constructs containing FLPe.

In order to create multiple gene deletions within the same parasite, it is critical that after the action of FLPe mutant parasites must regain sensitivity to the drugs used during selection. This can only be achieved if the *hdhfr* selection marker is completely absent from the parasite genome and that the FLPe/*bsd* containing plasmid is lost from the parasites after release of the drug pressure. The loss of these plasmids is thought to happen rapidly in parasites after the release of blasticidin pressure due to uneven segregation of such DNA elements into daughter merozoites. However, it has been reported that parasites can spontaneously acquire blasticidin resistance when exposed to sustained blasticidin treatment independent of the *bsd*-selectable marker [30]. We therefore tested the sensitivity of blood stages to both blasticidin and WR99210 using standard drug-susceptibility assays. We demonstrate that parasites have not acquired blasticidin resistance (Figure 2A). In addition the Δp5236gfp*FLPe parasites had regained the sensitivity to WR99210 after the recombinase treatment (Figure 2B).

### Generation of a generic gene-deletion construct containing FRT sites

We have generated a ‘standardised’ FRT gene-deletion construct, which contains FRT sites next to the gene targeting regions. These gene targeting regions can easily be exchanged for any gene of interest (see Figure S1C). This generic construct is an adapted version of the standard construct pHHT-FCU (see Material and Methods for construct details) that has two *P52* target regions introduced into *Sac*II/*Hpa*I digested pHHT-FCU (5′ -target region) and into *Nco*I/*Eco*RI digested pHHT-FCU (3′-target region). The two FRT sites reside just next to the targeting regions and flank the selectable marker. Each target region contains 4 unique restriction sites; for the 5′target region *Bsi*WI/*Mlu*I, *Bss*HII/*Sac*II and for the 3′target region *Nco*I/*Nhe*I, *Kpn*I/*Xma*I (Figure S1C).

## Discussion

In the absence of efficient forward genetic screens in malaria research, the targeted deletion/mutation of *Plasmodium* genes is now one of the most important methodologies to study the function of malaria parasite genes. However, the low efficiency of targeted gene deletion, the slow process of selecting gene deletion mutants and the limited number of drug-resistance markers greatly limits the analysis of *Plasmodium* genes. This analysis is of particular importance as more than 50% of *Plasmodium* genes have no homologs in other species (annotated ‘unknown function’) and the proteins encoded by a number of these genes maybe attractive targets for drugs or vaccines. To date there are no reported *P. falciparum* mutants that have had multiple, non-neighbouring, genes deleted. Here we describe a method of generating gene deletions in *P. falciparum* that makes use of the yeast FLP-recombinase enzyme to remove introduced resistance-markers and other plasmid DNA sequences from the mutants. This methodology facilitates the generation of multiple gene deletions or gene mutations in *P. falciparum* which is important in uncovering *Plasmodium* specific functions and processes. Moreover, this technique facilitates the generation of genetically attenuated parasites (GAP) permitting the removal of multiple genes. Gene deletion mutants of human malaria parasites, which completely arrest during their development inside hepatocytes, are currently being intensely investigated as potential whole-organism malaria vaccines[10], [20], [21].

**Figure 3:**
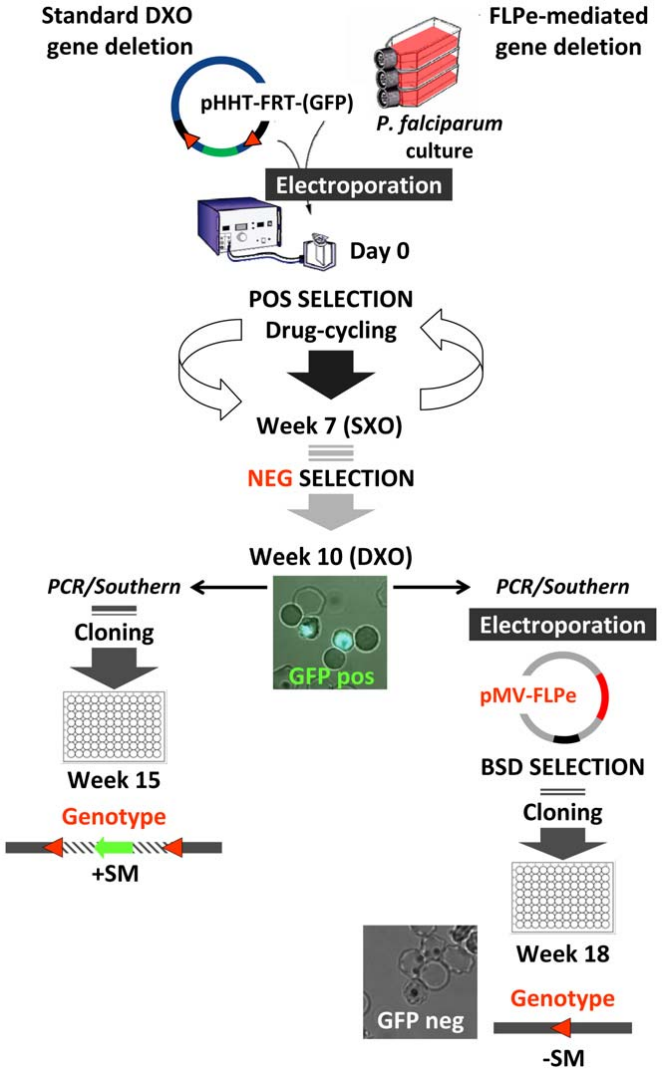
Schematic representation of the generation of FLPe-mediated ‘resistance marker-free’ *P. falciparum* mutants. Standard gene deletion by double cross-over (DXO) homologous recombination (left hand side) is compared to gene deletion using the FLPe-recombinase method described in this paper (right hand side). Both methods are essentially identical up to 10 weeks. First transformed parasites are treated by on/off cycling with the antimalarial drug WR99210 (POS SELECTION) to select for mutant parasites where the plasmid has become integrated into the genome by single cross-over (SXO) homologous recombination. Next negative drug selection (NEG SELECTION) using the drug 5-FC is applied to select for those parasites where an internal recombination (DXO) between plasmid and genomic sequences has occurred and the target gene is deleted. At this stage all transformed parasites are GFP positive as the *hdhfr*-resistance marker is fused to GFP. At this point conventional DXO gene deletion parasites are cloned by a method of limiting dilution. At week 15 cloned parasites still containing the resistance marker (+SM; shown in the standard DXO genotype schematic as a green arrow) can be expanded. In the FLPe recombinase method the gene deletion mutants selected after positive/negative selection are not cloned but immediately transformed with a plasmid encoding the enhanced FLP recombinase (pMV-FLPe). This plasmid is maintained episomally through blasticidin selection (BSD SELECTION) for one week after which BSD selection is released and once these parasites are detected in culture they are cloned by limiting dilution. At week 18, only 3 weeks longer than standard method, these resistance marker–free parasites can be expanded. Removal of the resistance marker is confirmed by the absence of GFP-expression as recombination between the introduced FRT sites (red triangles) has occurred removing plasmid, *gfp* and drug resistance marker sequences (-SM).

An advantage of the high efficiency of removal of FRTed sequences (>99%) from the mutant parasite genome by FLPe is that it is not necessary to clone the parasites before transfection with the FLPe-plasmid, thereby reducing the time for generation of the desired mutants. Consequently, the whole procedure of generating a gene-deletion mutant without resistance marker takes 18 weeks as compared to 15 weeks it currently takes to generate (double cross-over) gene deletion mutants with a selectable marker. In Figure 3 we show a schematic representation detailing and comparing the standard gene deletion with the FRT/FLPe deletion-recycling method described in this paper (Figure 3).

The use of the FLP/FRT system for gene removal in *Plasmodium* has been previously reported for the rodent parasite, *P. berghei.* However, this strategy permits deletion of genes only during development of the parasite in the mosquito. The method also consists of inserting FRT sites around the locus of interest in a parasite that expresses FLP recombinase driven from a mosquito stage-specific promoter[17], [18], [19]. The system makes use of either FLP or a low-activity FLP enzyme, termed FLPL. The activity of FLPL is greatly reduced at 37°C and maintained at this reduced level at 20°C–25°C, temperatures permissive for parasite development in the mosquito. Because this strategy to delete genes requires passage of the parasites through mosquitoes it will be extremely difficult to adapt this methodology to *P. falciparum*. Attempts to adapt the FLP or FLPL based system to delete genes during blood stage development have so far been unsuccessful. Analysis in blood stage of *P. berghei* only very low recombination efficiencies have been observed after prolonged cultivation in either FLP- or FLPL-expressing parasites at temperatures ranging from 21–37°C (personal observations; M.R. van Dijk and A.P. Waters *personal communication*). This low level of excision in *P. berghei* blood stages mediated by FLP or FLPL was comparable to that what we have observed with FLP-based excision of FRTed sequences in *P. falciparum* blood stages. The strong increase in recombination (>99%) observed with FLPe indicates that the use of the 37°C variant of FLP (i.e. FLPe) is the most important adaptation permitting efficient excision of heterologous DNA sequences. Interestingly we found that both FLP and FLPe had an ‘off target’ effect on blood stage development in culture, resulting in a reduced growth rate and/or arrest in parasite development. However, a beneficial side-effect of the growth delay of parasites containing FLPe-episomes is that those parasites that lose the FLP-plasmid after removal of BSD selection will outgrow the parasites that still retain FLPe-episomes. This then results in the enhancement of selection of episome-free parasites with the resistance marker removed. Indeed, we were unable to detect the FLPe construct by PCR at 3 weeks after the removal of BSD selection (data not shown).

We are now using the Δ*p5236* parasites generated in this study as the basis for introducing additional marker-free gene deletions in order to generate a GAP-vaccine that is not only potent but also safe for human use, specifically GAPs that are compromised at multiple points of development (multiple-deletions) and without the addition of heterologous sequences (i.e. no resistance markers). The procedures of efficient removal of drug-resistance markers in gene deletion mutants that do not affect either gametocyte production or drug-sensitivity, demonstrate that the FLPe recombinase system is an effective and powerful tool that offers new opportunities for *P. falciparum* transgenesis, both for the analysis of gene function and for the generation of genetically attenuated parasites - making the removal of resistance genes and multiple gene-deletion mutants possible.

While this manuscript was under review, a very similar method to re-cycle drug selectable markers in *P. falciparum* was published. In this study O'Neill and colleagues demonstrated that highly efficient site-specific recombination, removing introduced DNA, was obtained using Cre recombinase and loxP sites[31]. Interestingly, they observed very low levels of recombination, as did we, with standard (30°C optimal) FLP recombinase but do not report testing a 37°C optimised variant of FLP.

## Material and Methods

### Culture of *P. falciparum* blood stages and parasite cloning

Blood stages of *P. falciparum* parasites of line NF54 (wild-type; WT) and the different mutants generated in this study (see below) were cultured using *in vitro* culture conditions for *P. falciparum* previously described[32], [33], [34]. Subcultures of the different lines were established in the same semi-automated culture system. Fresh human red blood cells were obtained from Dutch National blood bank (Sanquin Nijmegen, NL; permission granted from donors for the use of blood products for malaria research), washed in serum free medium, and these were added to these cultures at parasitemias between 2–7%, thereby reducing the parasiteamia to 0.5% while maintaining a 5% hematocrit. Induction of gametocyte production in these cultures was performed as previously described[32], [33], [34].

Cloning of transgenic parasites was performed by the method of limiting dilution in 96 well plates[35]. Parasites of the positive wells were transferred to the semi-automated culture system and cultured for further phenotype and genotype analyses (see below).

### Generation of DNA constructs

The *pf52 and pf36* genes (PFD0215c and PFD0210c) of *P. falciparum* were disrupted using an adapted version of the standard construct (pHHT-FCU) for gene deletion using positive/negative selection procedure[9]. The pHHT-FRT-Pf5236 targeting construct was generated by inserting target sequences including FRT sites (in italic) for *p52* (primers BVS25: CATGCAATT*G* *aagttcctattctctagaaagtataggaacttc*aattcacaagcaactaaaatcaatatcc; *1638: CATGCCATGGtttgaataagttttacaacctgc) digested with MfeI and NcoI and p36 (primers BVS18: GAATTCGATATCgaagttcctatactttctagagaataggaacttccactcgaatgtgggatggcatcc*; 2589 (tccccgcggATGAGGTACATTCTCAGGAATC) digested with *Eco*RV and *Sac*II into the *Eco*RI, *Nco*I and *Hpa*I, *Sac*II sites respectively of pHHT-FCU. For construction of pHHT-FRT-(GFP)-Pf5236 the *hdfr* resistance gene was replaced by cloning the *Hin*dIII and *Sac*I digested *hdfr-gfp* fusion gene fragment from plasmid pBKHGint (Christian Flueck, unpublished) into *Hin*dIII and *Sac*I digested pHHT-FRT-Pf5236.

Two plasmids were generated that contain an FLP recombinase under the control of the *hsp86* promoter and the *blasticidin-S-deaminase* (*bsd*) resistance marker. The first construct, pMV-FLP was constructed by inserting the standard FLP gene as a 1322 bp fragment PCR amplified from plasmid FLP@UIS4 (kindly provided by Robert Menard, Pasteur Institute, Paris, France) using primers BVS53 (5′-GGTCCTCGAGatggtttccctttccc) and BVS54 (5′-TCGCCTCGAGttatatgcgtctatttatgtaggatg), into *Xho*I digested pHHT-FCU replacing the *fcu* open reading frame and subsequently cloning the *Not*I/SacII HSP86-FLP-pBDT fragment into the *Not*I/SacII digested pBSII-KS+ plasmid (Stratagene). The *bsd*-cassette (5′HRPIII-BSD-3′HRPII) derived from pCMB-BSD[7] was introduced in this plasmid through *Kpn*I/*Pst*I cloning of the 2800 bp 5′HRPIII-BSD-3′HRPII fragment, resulting in plasmid pMV-FLP. The second construct, pMV-FLPe, was constructed by inserting the standard FLPe (a 37°C thermostable enhanced allozyme of FLP recombinase[27]) gene as a 1340 bp fragment PCR amplified from plasmid pGaggs-FLPe (obtained via Addgene; www.addgene.org) using primers BVS120 (5′- gggtcgacAGATCTCACCATGGCTCCCAAGAAGAAGAGG) and BVS121 (5′- gggtcgacCTCGACTCTAGATCATTATATGCG) into the *Xho*I digested pMV-FLP plasmid, resulting in plasmid pMV-FLPe.

A generic construct containing FRT sites was made in which target regions are easily exchanged to target any gene of interest. The construct is an adapted version of the standard construct (pHHT-FCU; see above for construct details) in which *P52* target regions including FRT sites were introduced (5′*p52* with primer bvs29 (5′agcatgCCGCGGCGCGCTGCCAGAATGTTCTTGTTCG) and bvs30 (5′ CATGGTTAACGAAGTTCCTATACTTTCTAGAGAATAGGAACTTCGTACGCGTgcctttgttaatcaaagtaatccaaccg) into *Sac*II/*Hpa*I digested pHHT-FCU and for 3′*p52* primer bvs31(5′ agcatgGAATTCGAAGTTCCTATTCTCTAGAAAGTATAGGAACTTCccgggtaccCATATATTATATGTTCCTCTTG) and bvs32(5′ AGCATGCCATGGCTAGCcatacactttttctcatgag) into *Nco*I/*Eco*RI digested pHHT-FCU). Each target region contains 4 unique restriction sites for the 5′target region *Bsi*WI/*Mlu*I, *Bss*HII/*Sac*II and for the 3′target region *Nco*I/*Nhe*I, *Kpn*I/*Xma*I (SOM Fig. 1C). A *P52-*FRT containing generic construct and pMV-FLPe are available on request for research purposes.

DNA fragments were amplified by PCR amplification (Phusion, Finnzymes) from genomic *P. falciparum* DNA (NF54 strain) or from the described plasmids and all PCR fragments were sequenced after TOPO TA (Invitrogen) sub-cloning.

### Transfection and selection of transgenic parasites

Transfection of blood-stage parasites was performed as described[25] using a BTX electroporation system. Transfected parasites were cultured in a semi automated culture system. Selection of gene deletion mutants by positive and negative selection procedures were performed as described[9]. Transfection of gene deletion mutants with constructs containing FLP or FLPe (plasmids pMV-FLP, pMV-FLPe) and selection of blasticidin resistant parasite populations was performed as described[7].

### Genotype analysis of transgenic parasites

Genotype analysis of transformed parasites was performed by Expand Long range dNTPack (Roche) diagnostic PCR (LR-PCR) and Southern blot analysis. Genomic DNA of blood stages of WT or mutant parasites was isolated as described[36] and analyzed by LR-PCR using primer pair (p1, p2) 3258 (5′-TAAACCTATTTGAAGCTTTATAC) and 3259 (5′-CTTGTGGGAAATTACAATGAC) for correct integration of construct *p5236FRT* in the *pf52/36* locus by double cross over integration. The LR-PCR program has an elongation step of 62°C[26] for 10 minutes, and an annealing step of 48°C for 30 seconds. All other PCR settings were according to manufacturers instructions.

For Southern blot analysis, genomic DNA was digested with *Eco*RI/*Hin*dIII or with *Bcl*I for analysis of disruption of *pf52* and *pf36* respectively. DNA was size fractionated respectively on a 0.7% or 1% agarose gel and transferred to a Hybond-N membrane (Amersham) by gravitational flow[36]. The blot was pre-hybridized in Church buffer[37] followed by hybridization to a *pf52* and *pf36* specific probes (pHHT-FRT-Pf5236 or pHHT-FRT-(GFP)-Pf5236 digested with *Nco*I/*Xba*I (1089 bp) or *Sac*II/*Xba*I (902 bp) respectively) constituting the sequences used as target sequences for integration (see above). Both probes were labelled using the High Prime DNA labelling kit (Roche) and purified with Micro Biospin columns (Biorad).

### Fluorescence microscopy

Samples (2 µl) of infected red blood cells from cultures with parasitemias between 2 and 10% were incubated with Hoechst 33258 (10 µM) for 20 minutes at 37°C before mounting on a sealed cover slip slide. Hoechst- and GFP-fluorescence were analysed using a Zeiss Fluorescence microscope (1000x magnification) and Axiovision software.

### Gametocyte and male gamete production

Gametocyte production was established in cultures at day 13-15 after start of the gametocyte cultures by counting the number of mature gametocytes (stage II and stages IV/V) in Giemsa stained thin blood films[32]. Male gamete formation was determined by activation of exflagellation. Samples of 10 µl were taken from the cultures, infected red blood cells pelleted by centrifugation and resuspended in 10 µl of Foetal Calf Serum (pH 8.0) at room temperature for 10 minutes and then mounted on a cover slip. Exflagellation centers were counted under the light-microscope in 5 homogeneous fields of a single cell layer of red blood cells at a 400x magnification. The samples were scored as follows: if the mean number of exflagellation centers was >10/field they were scored as ++; <10/field they were scored as +; and none was scored as 0.

### Drug sensitivity assays

Drug sensitivity was analyzed as described[38] with some modifications. Briefly, infected blood cells (1% parasitemia) were cultured using the Candle Jar method in 24 wells culture plates containing serial drug dilutions of either WR99210[25] (Jacobus Pharmaceutical Company) or blasticidin[7] (Invitrogen). Medium was changed daily. The parasitemia in all culture wells was determined 96 hours after the start of the cultures by counting infected erythrocytes in Giemsa stained thin blood smears. The non-linear regression function for sigmoidal dose-response (variable slope) of the GraphPad Prism software is used to calculate the (best-fit) inhibitory concentration (IC_50_) values.

## Supporting Information

Figure S1 **(A) FLP/FLPe recombinase containing construct.** The construct for transient expression of standard FLP recombinase (plasmid pMV-FLP) or its 37°C thermostable enhanced allozyme, FLPe (plasmid pMV-FLPe). The *flp* and *flpe* genes are under the control of the *hsp80* promoter. These plasmids contain the *blasticidin-s-deaminase* (*bsd)* gene under control of the *hsp86* promotor. *hrp:* histidine rich protein; *pbdt*: *P.berghei dhfr* terminator. **(B) Delayed growth phenotypes of FLPe expressing blood stages in subcultures.** Growth of blood stages of wild type and mutant parasites in the presence or absence of FLPe expression in subcultures, showing a delayed growth phenotype in the presence FLPe expression. Solid arrows: Dilution of Δ*p5236gfp* subculturing to 0.5% parasiteamia with fresh erythrocytes. Dashed arrow: Dilution of WT*FLPe subculture with fresh erythrocytes. **(C) Generic pHHT-FRT-(GFP)-Pf52 construct.** The construct (pHHT-FRT-(GFP)-Pf52) for targeting deletion of the *p52* gene contains the two FRT sequences (red triangles) that are recognized by FLP. Indicated are the restriction sites that are introduced to facilitate exchange of *p52* targeting regions with targeting regions of other genes of interest. Each target region contains 4 unique restriction sites for the 5′target region *Bsi*WI/*Mlu*I, *Bss*HII/*Sac*II and for the 3′target region *Nco*I/*Nhe*I, *Kpn*I/*Xma*I. *cam*: calmodulin; *hrp*: histidine rich protein*; hsp:* heatshock protein*; fcu*: cytosine deaminase/uracil phosphoribosyl-transferase; *pbdt*: *P.berghei dhfr* terminator.
(TIF)

## References

[1] Gardner MJ, Hall N, Fung E, White O, Berriman M (2002). Genome sequence of the human malaria parasite Plasmodium falciparum.. Nature.

[2] Hall N, Karras M, Raine JD, Carlton JM, Kooij TW (2005). A comprehensive survey of the Plasmodium life cycle by genomic, transcriptomic, and proteomic analyses.. Science.

[3] Carlton JM, Angiuoli SV, Suh BB, Kooij TW, Pertea M (2002). Genome sequence and comparative analysis of the model rodent malaria parasite Plasmodium yoelii yoelii.. Nature.

[4] Pain A, Bohme U, Berry AE, Mungall K, Finn RD (2008). The genome of the simian and human malaria parasite Plasmodium knowlesi.. Nature.

[5] Balu B, Adams JH (2007). Advancements in transfection technologies for Plasmodium.. Int J Parasitol.

[6] Duraisingh MT, Triglia T, Cowman AF (2002). Negative selection of Plasmodium falciparum reveals targeted gene deletion by double crossover recombination.. Int J Parasitol.

[7] Mamoun CB, Gluzman IY, Goyard S, Beverley SM, Goldberg DE (1999). A set of independent selectable markers for transfection of the human malaria parasite Plasmodium falciparum.. Proc Natl Acad Sci U S A.

[8] Wu Y, Kirkman LA, Wellems TE (1996). Transformation of Plasmodium falciparum malaria parasites by homologous integration of plasmids that confer resistance to pyrimethamine.. Proc Natl Acad Sci U S A.

[9] Maier AG, Braks JA, Waters AP, Cowman AF (2006). Negative selection using yeast cytosine deaminase/uracil phosphoribosyl transferase in Plasmodium falciparum for targeted gene deletion by double crossover recombination.. Mol Biochem Parasitol.

[10] Vaughan AM, Wang R, Kappe SH (2010). Genetically engineered, attenuated whole-cell vaccine approaches for malaria.. Hum Vaccin.

[11] Aly AS, Downie MJ, Mamoun CB, Kappe SH (2010). Subpatent infection with nucleoside transporter 1-deficient Plasmodium blood stage parasites confers sterile protection against lethal malaria in mice.. Cell Microbiol.

[12] Spaccapelo R, Janse CJ, Caterbi S, Franke-Fayard B, Bonilla JA Plasmepsin 4-deficient Plasmodium berghei are virulence attenuated and induce protective immunity against experimental malaria.. Am J Pathol.

[13] Ting LM, Gissot M, Coppi A, Sinnis P, Kim K (2008). Attenuated Plasmodium yoelii lacking purine nucleoside phosphorylase confer protective immunity.. Nat Med.

[14] Cfmpfhu, EMEA (2006). Guideline on environmental risk assessments for medicinal products consisting of, or containing, genetically modified organisms (GMOs)..

[15] Frey J (2007). Biological safety concepts of genetically modified live bacterial vaccines.. Vaccine.

[16] Andrews BJ, Proteau GA, Beatty LG, Sadowski PD (1985). The FLP recombinase of the 2 micron circle DNA of yeast: interaction with its target sequences.. Cell.

[17] Carvalho TG, Menard R (2005). Manipulating the Plasmodium genome.. Curr Issues Mol Biol.

[18] Carvalho TG, Thiberge S, Sakamoto H, Menard R (2004). Conditional mutagenesis using site-specific recombination in Plasmodium berghei.. Proc Natl Acad Sci U S A.

[19] Combe A, Giovannini D, Carvalho TG, Spath S, Boisson B (2009). Clonal conditional mutagenesis in malaria parasites.. Cell Host Microbe.

[20] van Schaijk BC, Janse CJ, van Gemert GJ, van Dijk MR, Gego A (2008). Gene disruption of Plasmodium falciparum p52 results in attenuation of malaria liver stage development in cultured primary human hepatocytes.. PLoS One.

[21] VanBuskirk KM, O'Neill MT, De La Vega P, Maier AG, Krzych U (2009). Preerythrocytic, live-attenuated Plasmodium falciparum vaccine candidates by design.. Proc Natl Acad Sci U S A.

[22] Gerloff DL, Creasey A, Maslau S, Carter R (2005). Structural models for the protein family characterized by gamete surface protein Pfs230 of Plasmodium falciparum.. Proc Natl Acad Sci U S A.

[23] Thompson J, Janse CJ, Waters AP (2001). Comparative genomics in Plasmodium: a tool for the identification of genes and functional analysis.. Mol Biochem Parasitol.

[24] van Dijk MR, Douradinha B, Franke-Fayard B, Heussler V, van Dooren MW (2005). Genetically attenuated, P36p-deficient malarial sporozoites induce protective immunity and apoptosis of infected liver cells.. Proc Natl Acad Sci U S A.

[25] Fidock DA, Wellems TE (1997). Transformation with human dihydrofolate reductase renders malaria parasites insensitive to WR99210 but does not affect the intrinsic activity of proguanil.. Proc Natl Acad Sci U S A.

[26] Su XZ, Wu Y, Sifri CD, Wellems TE (1996). Reduced extension temperatures required for PCR amplification of extremely A+T-rich DNA.. Nucleic Acids Res.

[27] Buchholz F, Angrand PO, Stewart AF (1998). Improved properties of FLP recombinase evolved by cycling mutagenesis.. Nat Biotechnol.

[28] Buchholz F, Ringrose L, Angrand PO, Rossi F, Stewart AF (1996). Different thermostabilities of FLP and Cre recombinases: implications for applied site-specific recombination.. Nucleic Acids Res.

[29] Gardiner DL, Dixon MW, Spielmann T, Skinner-Adams TS, Hawthorne PL (2005). Implication of a Plasmodium falciparum gene in the switch between asexual reproduction and gemetocytogenesis.. Mol Biochem Parasitol.

[30] Hill DA, Pillai AD, Nawaz F, Hayton K, Doan L (2007). A blasticidin S-resistant Plasmodium falciparum mutant with a defective plasmodial surface anion channel.. Proc Natl Acad Sci U S A.

[31] O'Neill MT, Phuong T, Healer J, Richard D, Cowman AF (2010). Gene deletion from Plasmodium falciparum using FLP and Cre recombinases: Implications for applied site-specific recombination.. Int J Parasitol.

[32] Ponnudurai T, Lensen AH, Meis JF, Meuwissen JH (1986). Synchronization of Plasmodium falciparum gametocytes using an automated suspension culture system.. Parasitology.

[33] Ifediba T, Vanderberg JP (1981). Complete in vitro maturation of Plasmodium falciparum gametocytes.. Nature.

[34] Ponnudurai T, Lensen AH, Leeuwenberg AD, Meuwissen JH (1982). Cultivation of fertile Plasmodium falciparum gametocytes in semi-automated systems. 1. Static cultures.. Trans R Soc Trop Med Hyg.

[35] Thaithong S, Panyim S, Wilairat P, Yuthavong Y (1985). Cloning of Malaria Parasites..

[36] Sambrook J, Russel WD (2001). Molecular Cloning: a laboratory manual..

[37] Church GM, Gilbert W (1984). Genomic sequencing.. Proc Natl Acad Sci U S A.

[38] Thaithong S, Beale GH (1981). Resistance of ten Thai isolates of Plasmodium falciparum to chloroquine and pyrimethamine by in vitro tests.. Trans R Soc Trop Med Hyg.

