# Supplementary figures and images for: Removal of Heterologous Sequences from Plasmodium falciparum Mutants Using FLPe-Recombinase
Source: PLoS One. 2010 Nov 30;5(11):e15121. doi: 10.1371/journal.pone.0015121 (PMC2994908; doi:10.1371/journal.pone.0015121)

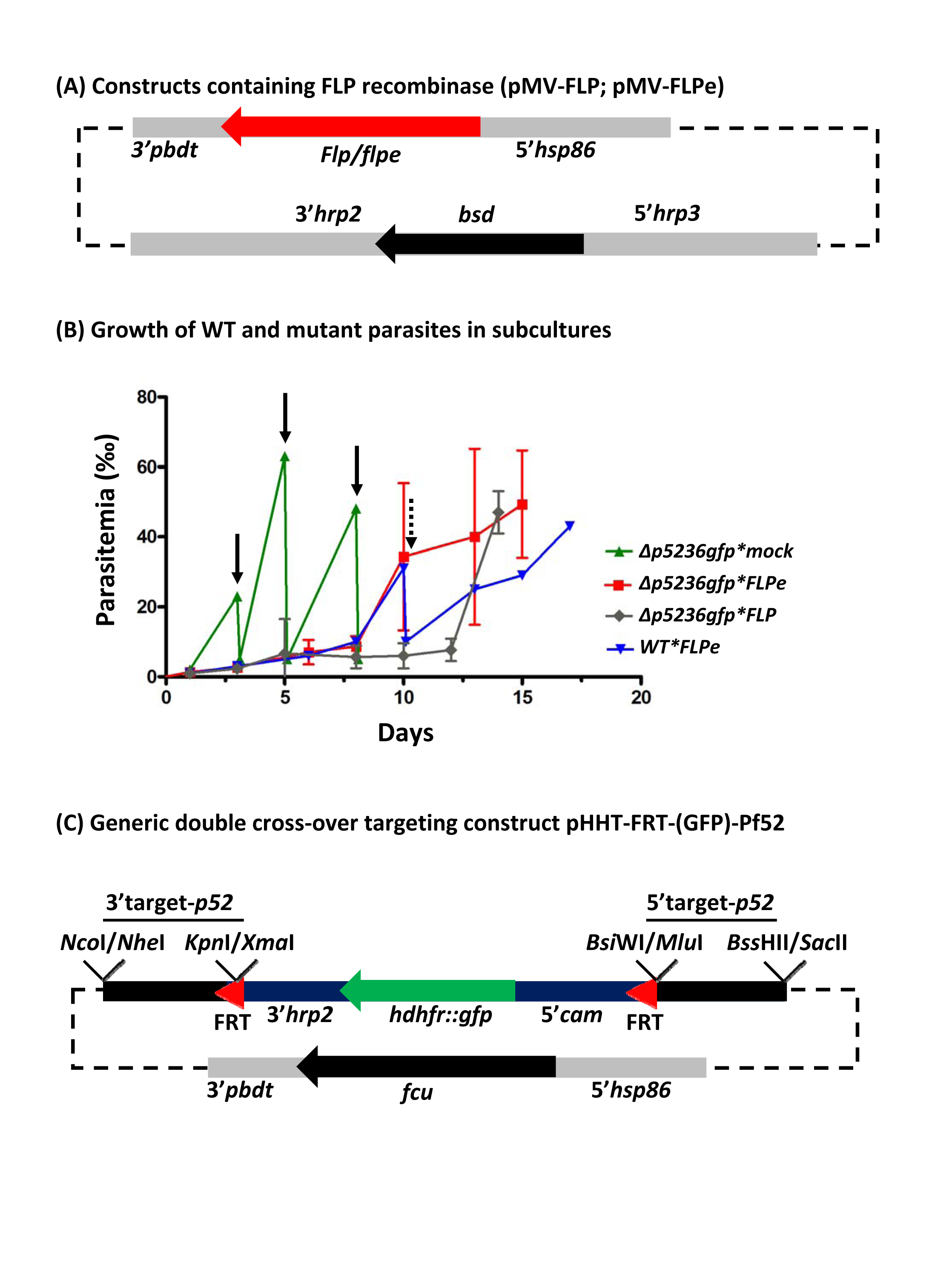

Supplement: Figure S1 — (A) FLP/FLPe recombinase containing construct. The construct for transient expression of standard FLP recombinase (plasmid pMV-FLP) or its 37°C thermostable enhanced allozyme, FLPe (plasmid pMV-FLPe). The flp and flpe genes are under the control of the hsp80 promoter. These plasmids contain the blasticidin-s-deaminase (bsd) gene under control of the hsp86 promotor. hrp: histidine rich protein; pbdt: P.berghei dhfr terminator. (B) Delayed growth phenotypes of FLPe expressing blood stages in subcultures. Growth of blood stages of wild type and mutant parasites in the presence or absence of FLPe expression in subcultures, showing a delayed growth phenotype in the presence FLPe expression. Solid arrows: Dilution of Δp5236gfp subculturing to 0.5% parasiteamia with fresh erythrocytes. Dashed arrow: Dilution of WT*FLPe subculture with fresh erythrocytes. (C) Generic pHHT-FRT-(GFP)-Pf52 construct. The construct (pHHT-FRT-(GFP)-Pf52) for targeting deletion of the p52 gene contains the two FRT sequences (red triangles) that are recognized by FLP. Indicated are the restriction sites that are introduced to facilitate exchange of p52 targeting regions with targeting regions of other genes of interest. Each target region contains 4 unique restriction sites for the 5′target region BsiWI/MluI, BssHII/SacII and for the 3′target region NcoI/NheI, KpnI/XmaI. cam: calmodulin; hrp: histidine rich protein; hsp: heatshock protein; fcu: cytosine deaminase/uracil phosphoribosyl-transferase; pbdt: P.berghei dhfr terminator. (TIF) [file pone.0015121.s001.tif]
